# Supplementary material for: Improved genetically encoded near-infrared fluorescent calcium ion indicators for in vivo imaging
Source: PLoS Biol. 2020 Nov 24;18(11):e3000965. doi: 10.1371/journal.pbio.3000965 (PMC7723245; doi:10.1371/journal.pbio.3000965)
Supplement: S1 Table — (DOCX) [file pbio.3000965.s001.docx]

**Supporting information**

**S1 Table. Spectral, photochemical and biochemical properties of NIR-GECO2G, NIR-GECO2, and NIR-GECO1.**

| Indicator | [Ca^2+^] (mM) | EC (× 1000 mM^-1^*cm^-1^) | QY (%) | p*K*_a_ | *K*_d_ (nM) | Hill Coeff. (n) | *k*_off_  (s^-1^) |
| --- | --- | --- | --- | --- | --- | --- | --- |
| NIR-GECO2G | 0 | 74 | 6.1 | 5.34 | 480 | 0.78 | 3.678 |
|  | 5 | 21 | 2.1 | 4.84 |  |  |  |
| NIR-GECO2 | 0 | 67 | 5.9 | 5.26 | 331 | 0.94 | 2.995 |
|  | 5 | 18 | 1.4 | 4.85 |  |  |  |
| NIR-GECO1 | 0 | 69 | 6.0 | 5.14 | 885 | 0.99 | 2.354 |
|  | 5 | 20 | 2.0 | 4.84 |  |  |  |

Abbreviations: EC, extinction coefficient; QY, quantum yield; p*K*_a_, pH corresponding to 50% of the maximal fluorescence brightness measured at optimal pH; *K*_d_, *K*_d_ for Ca^2+^; *k*_off_, Ca^2+^-dissociation kinetics measured by stopped-flow spectrometer.
