# Supplementary figures and images for: Improved genetically encoded near-infrared fluorescent calcium ion indicators for in vivo imaging
Source: PLoS Biol. 2020 Nov 24;18(11):e3000965. doi: 10.1371/journal.pbio.3000965 (PMC7723245; doi:10.1371/journal.pbio.3000965)

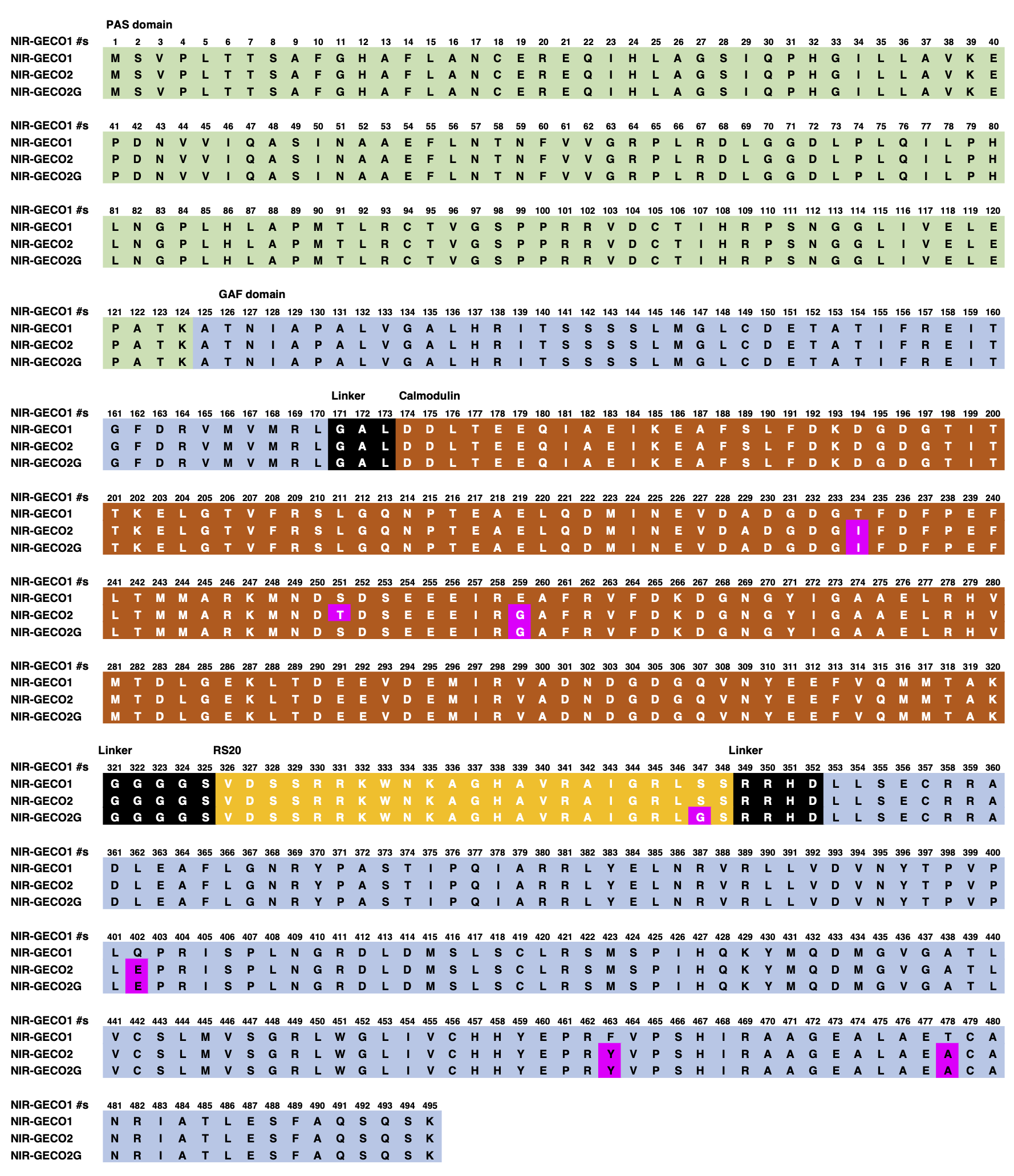

Supplement: S1 Fig — Single amino acid changes relative to NIR-GECO1 are highlighted with a magenta background. PAS domain, GAF domain, linkers, calmodulin, and RS20 are shown as light green, light blue, black, brown, and yellow, respectively. NIR, near-infrared. (TIFF) [file pbio.3000965.s002.tiff]

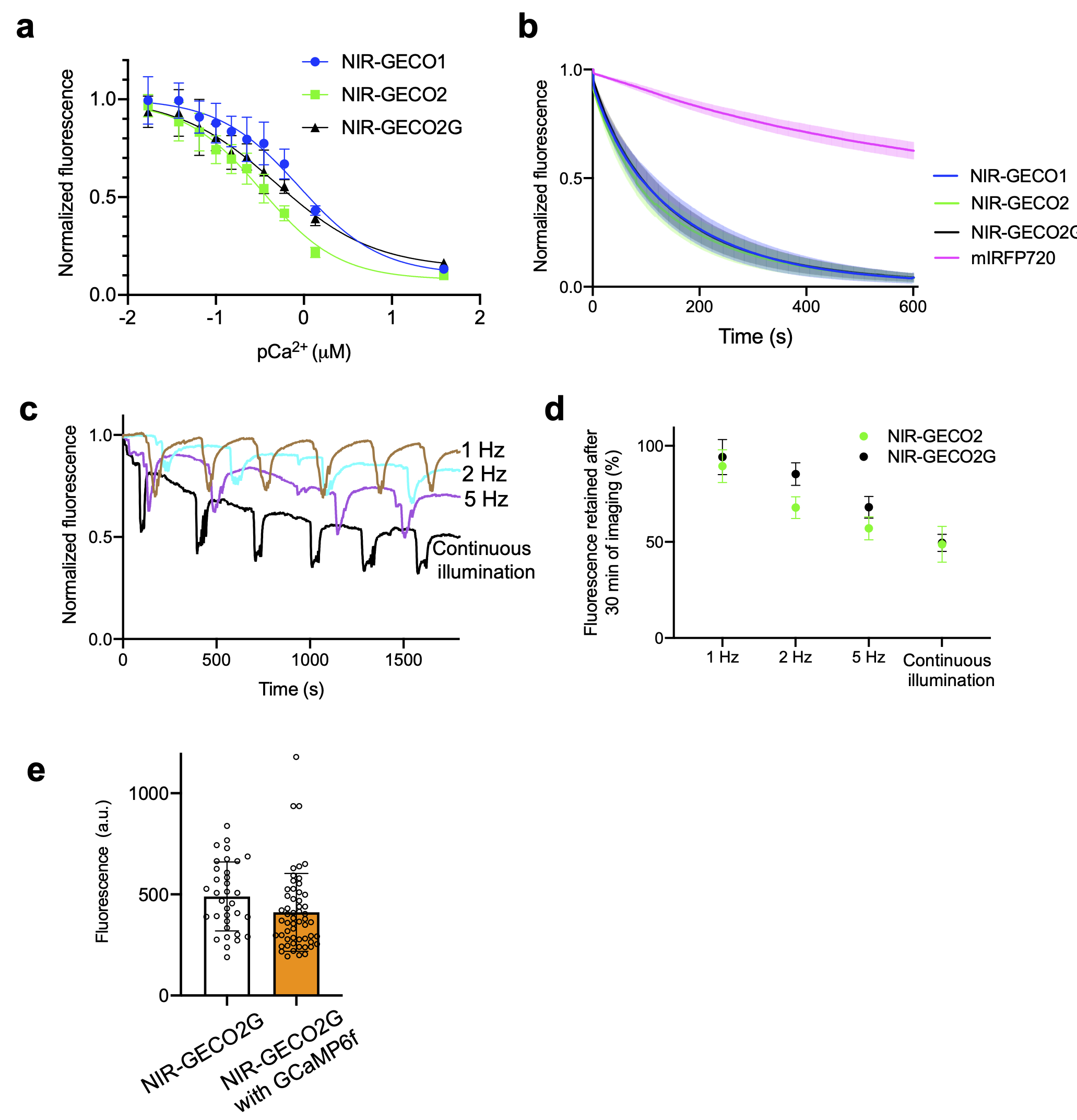

Supplement: S2 Fig — (a) Ca2+ titration curves of NIR-GECO1, NIR-GECO2, and NIR-GECO2G (center values are the mean and error bars are SD; n = 3). (b) Photobleaching curves of NIR-GECO variants and mIRFP720 (n = 11, 11, 16, and 9 neurons for NIR-GECO1, NIR-GECO2, NIR-GECO2G, and miRFP720, respectively). Mean value (solid line) and SD (shaded areas) are shown. Cells were continuously illuminated with 631/28 nm at 80 mW/mm2 during the experiment. Images were taken every 5 seconds. (c) Representative fluorescence curves of NIR-GECO2G with different imaging rates with 631/28-nm excitation at 2.8 mW/mm2 (this is the excitation intensity that we used for wield-field imaging; exposure time: 100 ms). (d) Quantitative data for the photobleaching of NIR-GECO2G and NIR-GECO2 in cultured cells with the same imaging conditions as in c. Data are shown as mean ± SD (NIR-GECO2G: n = 70, 71, 62, and 45 cells for 1 Hz, 2 Hz, 5 Hz, and continuous illumination, respectively; NIR-GECO2: n = 26, 36, 35, and 35 cells for 1 Hz, 2 Hz, 5 Hz, and continuous illumination, respectively). (e) Brightness of NIR-GECO2G and NIR-GECO2G with co-expression of GCaMP6f in neurons (n = 34 and 56 for NIR-GECOG and NIR-GECO2G with GCaMP6f, respectively; data are shown as mean ± SD). The underlying data for a, b, d, and e can be found in S1 Data. NIR, near-infrared; SD, standard deviation. (TIFF) [file pbio.3000965.s003.tiff]

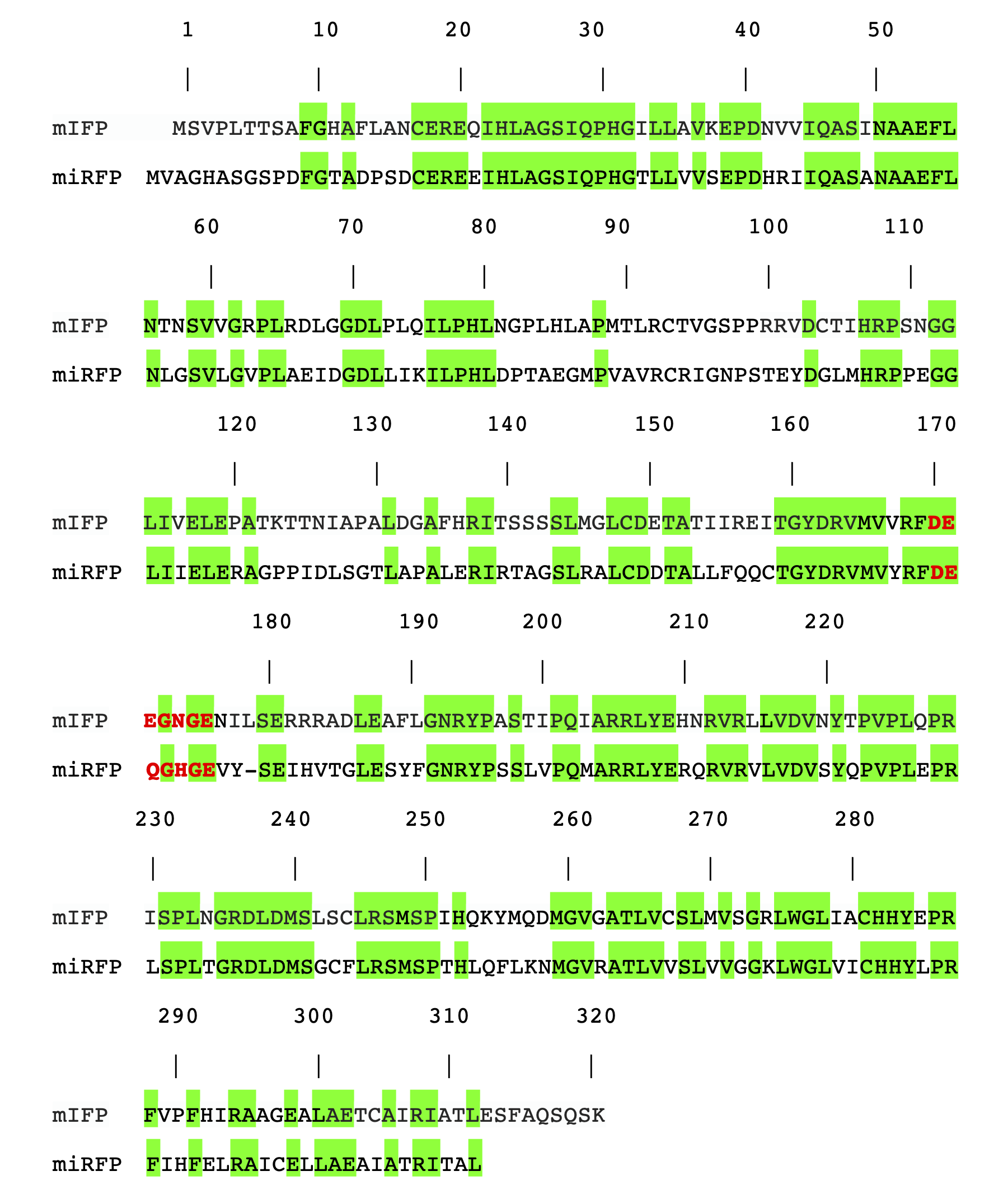

Supplement: S4 Fig — Alignment numbering is based on mIFP. The structurally analogous residues between mIFP and mIRFP are highlighted in green. Residues that were replaced by CaM-RS20 to make NIR-GECO1, and the prototype mIRFP-based Ca2+ indicator, respectively, are in bold and red. NIR, near-infrared. (TIFF) [file pbio.3000965.s005.tiff]

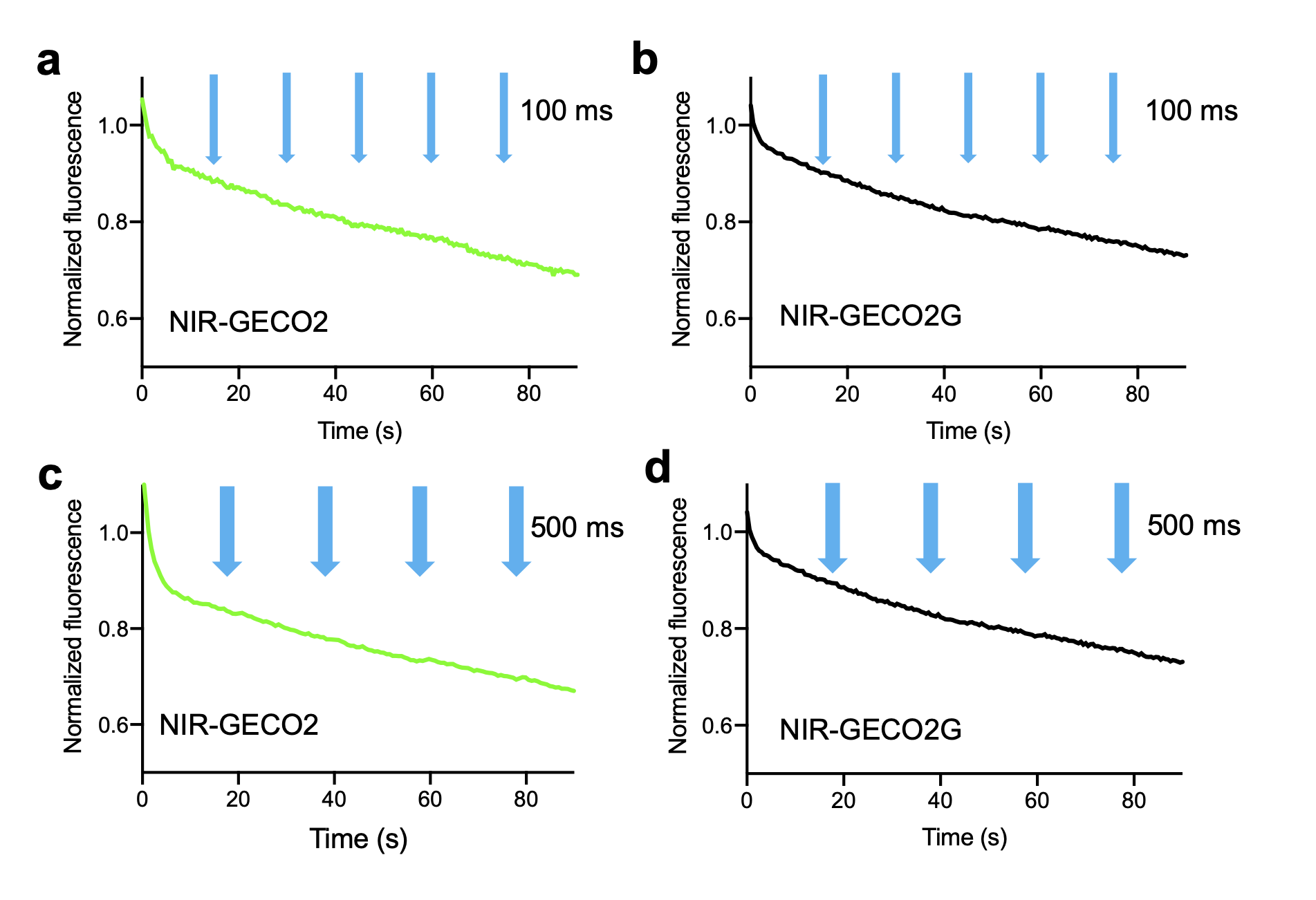

Supplement: S5 Fig — (a, b) Averaged fluorescence traces of NIR-GECO2 (a, n = 8 cells) and NIR-GECO2G (b, n = 9 cells) in response to 100-ms blue light illumination. (c, d) Averaged fluorescence traces of NIR-GECO2 (c, n = 9 cells) and NIR-GECO2G (d, n = 11 cells) in response to 500-ms blue light illumination. Illumination: 470 nm at 6.2 mW/mm2, which is 3.3-fold higher than the intensity used for the experiments in Fig 2. The underlying data for a to d can be found in S1 Data. NIR, near-infrared. (TIFF) [file pbio.3000965.s006.tiff]

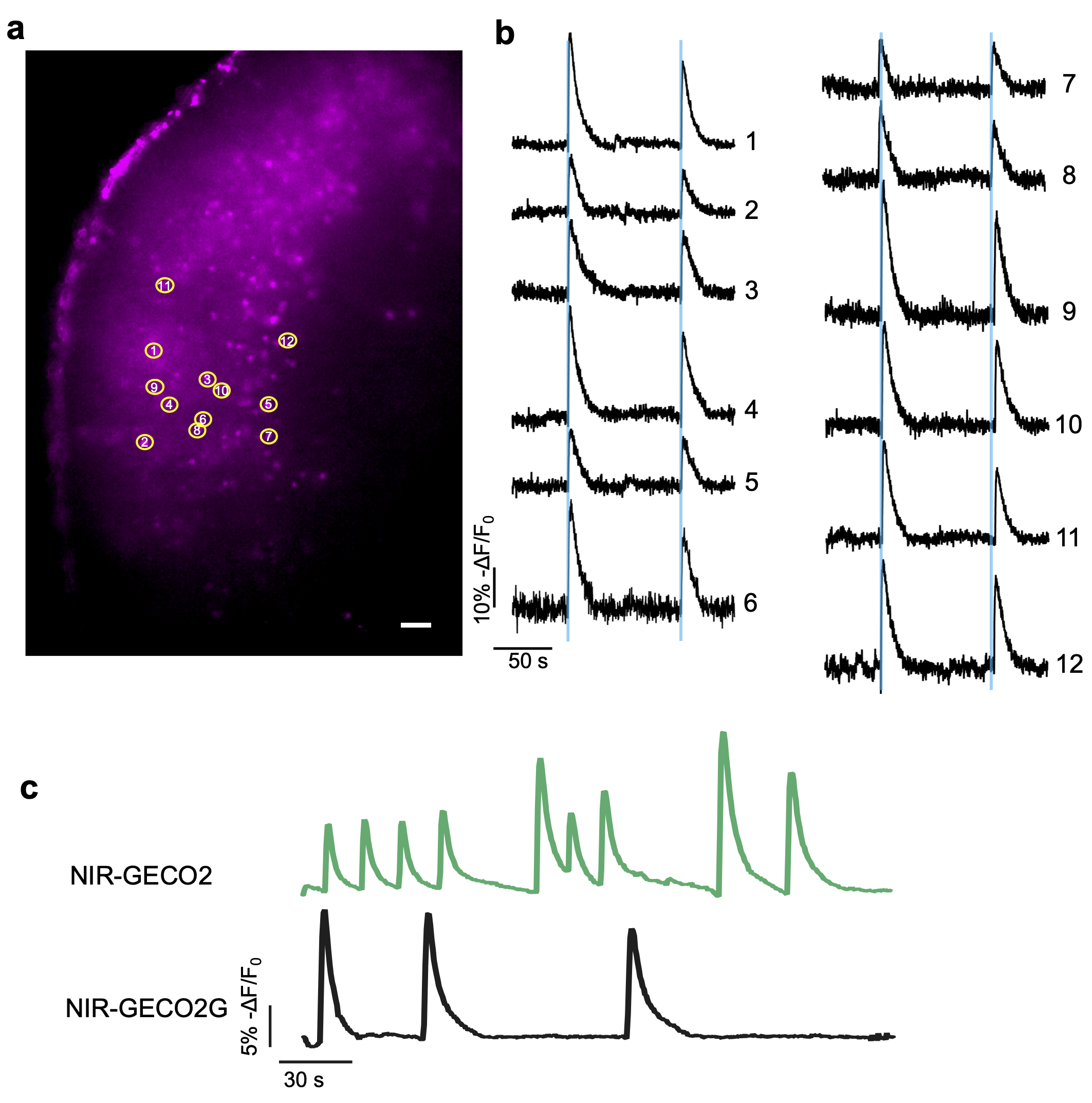

Supplement: S6 Fig — (a) Wide-field image of a mouse brain slice with co-expression of NIR-GECO2 and CoChR, fluorescence of NIR-GECO2 is shown (λex = 631/28 nm and λem = 664LP). Scale bar, 50 μm. (b) Fluorescence of NIR-GECO2 (acquisition rate 100 Hz) in response to 200-ms blue light stimulation (470/20 nm, 0.157 mW/mm2, indicated by blue bar). The numbers of the traces correspond to the neurons labeled in a. (c) Single-trial wide-field imaging of 4-aminopyridine (1 mM final concentration) evoked neuronal activity from the 2 representative neurons in brain slices expressing NIR-GECO2 and NIR-GECO2G, respectively (λex = 631/28 nm and λem = 664LP; acquisition rate: 10 Hz). NIR, near-infrared. (TIFF) [file pbio.3000965.s007.tiff]

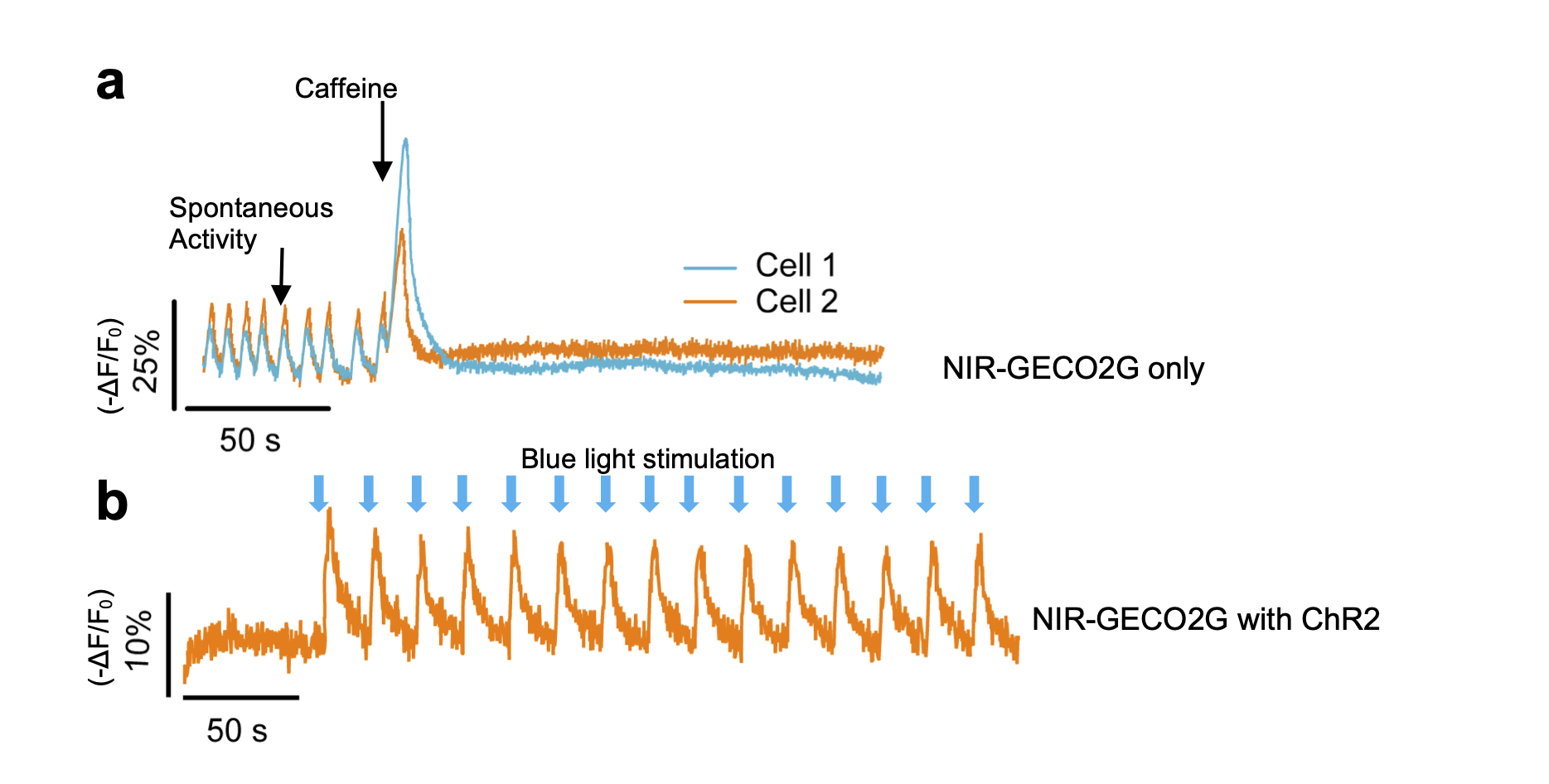

Supplement: S7 Fig — (a) Representative fluorescence recording of spontaneous and caffeine-evoked Ca2+ oscillations using NIR-GECO2G in iPSC-CMs. (b) Representative single-trial fluorescence recording of blue light-stimulated Ca2+ oscillations (470 nm at a power of 1.9 mW/mm2) using NIR-GECO2G in ChR2 expressed iPSC-CMs. iPSC-CM, induced pluripotent stem cell-derived cardiomyocyte; NIR, near-infrared. (TIFF) [file pbio.3000965.s008.tiff]

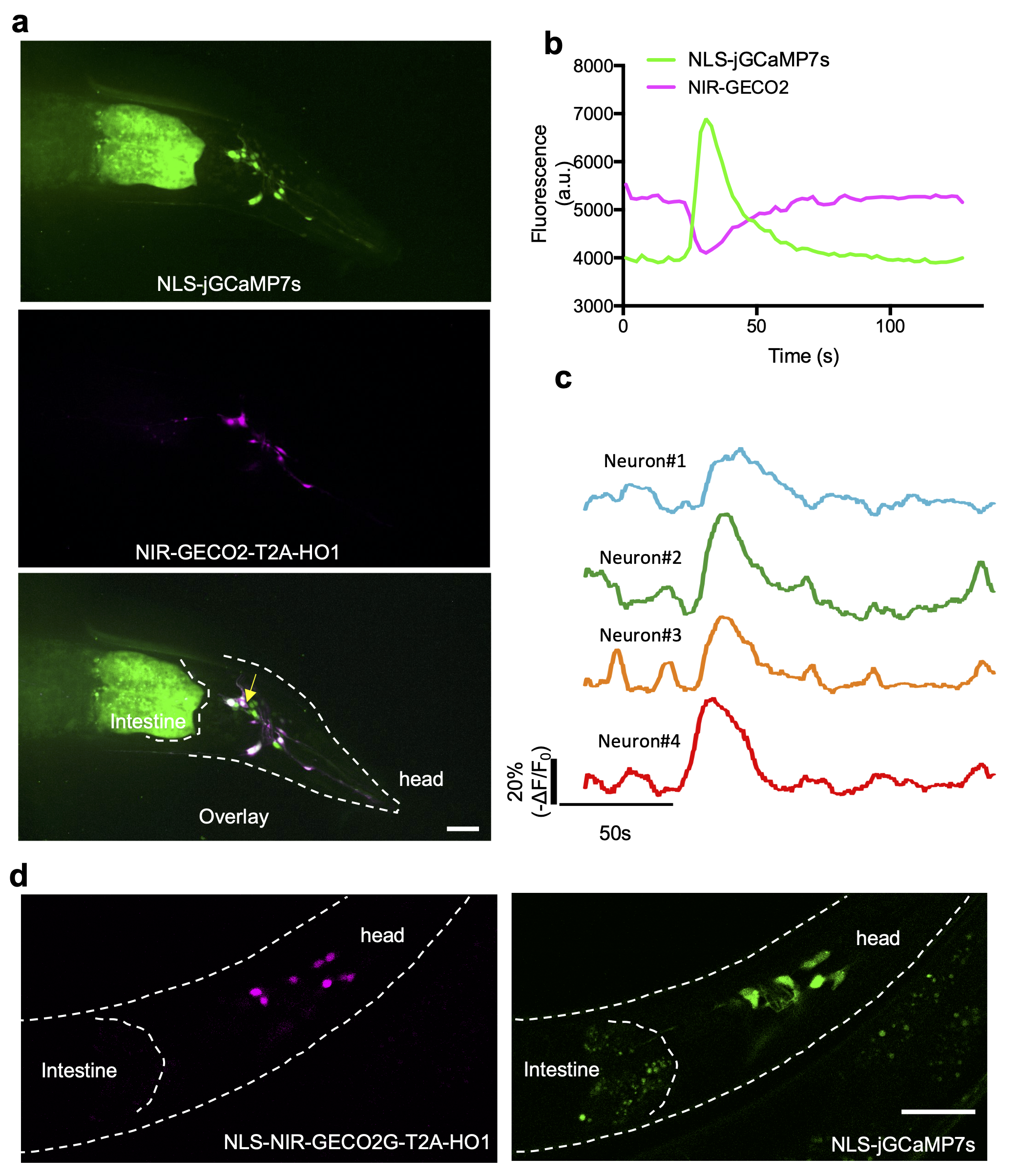

Supplement: S8 Fig — (a) Representative confocal images of worms co-expressing NIR-GECO2-T2A-HO1 and NLS-jGCaMP7s (representative of more than 3 worms). Top, fluorescent image of neurons expressing NLS-jGCaMP7s (λex = 488-nm laser light, λem = 527/50 nm). Middle, fluorescent image of neurons expressing NIR-GECO2-T2A-HO1 (λex = 640-nm laser light, λem = 685/40 nm). Bottom, overlay image of green channel and NIR channel. Scale bar, 25 μm. (b) Spontaneous Ca2+ fluctuation of a representative worm neuron (indicated in a by a yellow arrow) co-expressing NIR-GECO2 and NLS-jGCaMP7s. Imaging conditions were identical to the experiments in a, acquisition rate: 2 Hz. (c) Representative spontaneous Ca2+ oscillations of worm neurons reported by NIR-GECO2 (acquisition rate 2 Hz). Imaging conditions were identical to the experiments in a. (d) Representative confocal images of worms co-expressing NLS-NIR-GECO2G-T2A-HO1 (left) and NLS-jGCaMP7s (right). Imaging conditions were identical to the experiments in a. Representative of more than 3 worms. Scale bar, 25 μm. NIR, near-infrared. (TIFF) [file pbio.3000965.s009.tiff]

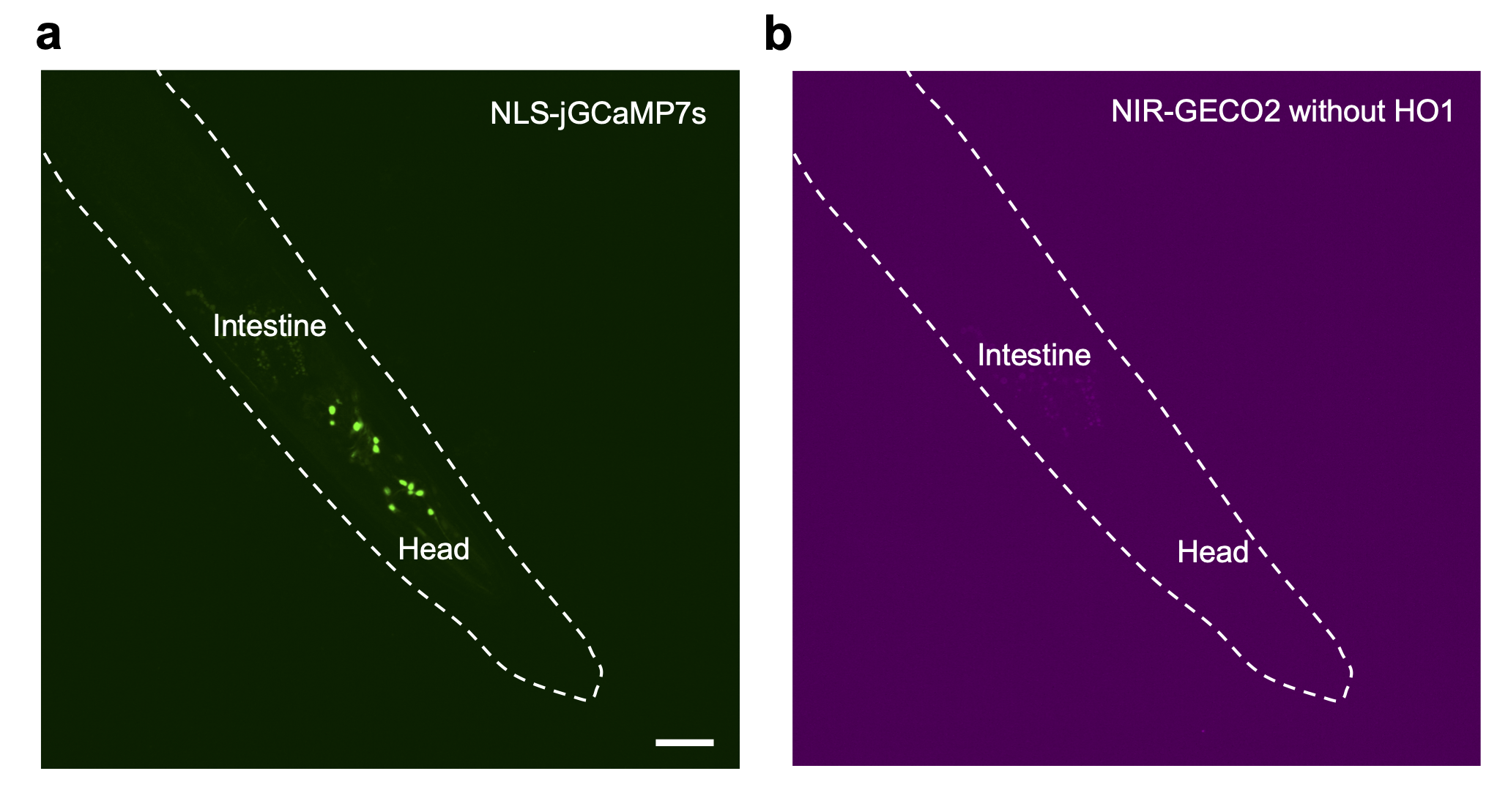

Supplement: S9 Fig — Scale bar, 25 μm; representative of n = 9 worms. NIR, near-infrared. (TIFF) [file pbio.3000965.s010.tiff]
