# Supplementary material for: Improved genetically encoded near-infrared fluorescent calcium ion indicators for in vivo imaging
Source: PLoS Biol. 2020 Nov 24;18(11):e3000965. doi: 10.1371/journal.pbio.3000965 (PMC7723245; doi:10.1371/journal.pbio.3000965)
Supplement: Attachment — Submitted filename: Response to the requirements from Editor.pdf [file pbio.3000965.s014.pdf]

ETHICS STATEMENT:

-- Please include in your manuscript the ID numbers of the protocols approved by the Massachusetts Institute of Technology Committee on Animal Care and by the Montreal Neurological Institute Animal Care Committee.

We now have included those protocol numbers in the Ethics Statement.

DATA POLICY:
Regardless of the method selected, please ensure that you provide the individual numerical values that underlie the summary data displayed in the following figure panels: Figures 1b-e, 2a-d, 3bce, 4a-e, S2abde, S3ab, and S5a-d.

NOTE: the numerical data provided should include all replicates AND the way in which the plotted mean and errors were derived (it should not present only the mean/average values).

Please also ensure that each figure legend in your manuscript includes information on where the underlying data can be found and that your supplemental data file/s has/have a legend.

Please ensure that your Data Statement in the submission system accurately describes where your data can be found.

We now have added file S1_Data.xlsx that includes the individual numeric data underlying Fig1b-e, 2a-d, 3bce, 4aa-e, S2abde, S3ab, and S5a-d. We also added a sentence at the end of each figure containing the information on where the underlying data can be found.
