# Supplementary material for: Improved genetically encoded near-infrared fluorescent calcium ion indicators for in vivo imaging
Source: PLoS Biol. 2020 Nov 24;18(11):e3000965. doi: 10.1371/journal.pbio.3000965 (PMC7723245; doi:10.1371/journal.pbio.3000965)
Supplement: Attachment — Submitted filename: NIR-GECO2 respond to reviewer comments V2.pdf [file pbio.3000965.s013.pdf]

REVIEWS:

Reviewer #1: In general, compared to the conventional fluorescent calcium ion optogenetic tools and indicators functioned under visible wavelength light irradiation, the NIR genetically-encoded counterparts provide great advantages of reduced light toxicity, less background cross-talk with visible-light as well as decreased scattering and absorption in mammalian tissues. However, several disadvantages including lower brightness and limited fluorescence response remained the concerns to hamper their further applications for imaging of neuronal activity in vitro and in vitro. To overcome these technical barriers, in this study, qiao and co-authors genetically encoded two ca2+ indicators, NIR-GECO2 and NIR-GECO2G, to provide substantial improvements of neuronal imaging of Ca2+ dynamics in cultured cells, mouse brain slices, and C. elegans and Xenopus laevis in vivo. Even though some new challenges like less brightness, slower kinetics, and faster photobleaching still need to be further improved. Apparently, the authors have carried out extensive experiments and the manuscript has been well prepared. The referee thus supports the publication of this interesting study in Plos Biology as the current stage.

Response: We appreciate the supportive comments and fully agree that there still remain challenges to be overcome and improvements to be realized.

Reviewer #2: In this manuscript by the Campbell lab (Qian et al), the authors described new variants of genetically encoded near-IR Ca2+ indicator NIR-GECO2 and NIR-GECO-2G. Compared to previously developed NIR-GECO1, there was not significant improvement in brightness or photo-stability, but they have 2-4 fold higher affinity to Ca2+. This increased dF / Fo of Ca2+ signals in neurons by 2-3 times. They showed that the new reagents are useful for Ca2+ imaging in acute slices, as well as in vivo imaging in C-elegans or Xenopus Laevis.

Overall the improvement in Ca2+ sensitivity seems to be significant, and thus the reagent should be useful for the community. One significant concern is the lack of control experiments for the photo-conversion by blue light illumination (Fig. 2). Since they used channel rhodopsin to test the sensitivity of NIR-GECO in these experiments, I think it is important to report to what degree NIR-GECO shows photo-conversion by blue light. For the community, the degree of compatibility with channel rhodopsin would be a great interest too.

Response: We appreciate the supportive comments and are confident that this improved tool will be useful to a broad community of researchers. We had previously confirmed that the first generation NIR-GECO1 indicator exhibited no artifactual fluorescence changes in response to blue light illumination. We have now also tested this for NIR-GECO2 and NIR-GECO2G and have added a new supplementary figure (Supplementary Fig. 5) with the results. According to our results, there was no detectable photoconversion for NIR-GECO2 and NIR-GECO2G in response to illumination with 470 nm light at 6.2 mW/mm^2.

Reviewer #3: Qian et al. reported development and in vivo demonstration of two improved near-infrared fluorescence calcium indicators. Compared to the parent sensor, NIR-GECO1, these two variants displayed increased affinity to calcium and about 3 to 4-fold higher ΔF/F in response to single-field stimuli in dissociated neuronal culture or light-stimuli (100ms) in the brain slice. Other intrinsic properties remain similar to NIR-GECO1. The improved sensitivity permitted populational imaging of optogenetically triggered or spontaneous neuronal activity in c.elegans and in Xenopus laevis, thus representing a substantial improvement compared to NIR-GECO1.
The scope and novelty are appropriate to PLOS Biology. However, the authors should address/discuss a few points about in vivo utility of these sensors.

Response: Thank you for the supportive comments and suggestions.

1. One of potential advantages using NIR-based probes is the depth penetration. In Fig 3 and 4, the authors nicely demonstrated calcium signals detected by NIR-GECO. It will significantly strengthen the utility of these probes if the authors compare the signal-to-noise ratio of NIR-GECO and jGCaMP/GCaMP and 3D volume/optical dissection depth possibly increased by using longer-wavelength.

Response: Thank you for the valuable suggestions. To address this suggestion, we have performed new imaging experiments in *C. elegans* and we present the results in a new figure (new Fig. 4; original Fig.4 is now Fig. 5) that provides a comparison of the fluorescence intensity, signal-to-background ratio (SBR), and signal-to-noise ratio (SNR) between NIR-GECO2 and jGCaMP7s. These experiments revealed that the fluorescence intensity of NIR-GECO2 was about 1.5-fold higher than that of jGCaMP7s in neurons at resting state, the SBR of NIR-GECO2 was ~5-fold larger than that of jGCaMP7s, and the SNRs of NIR-GEO2 and jCaMP7s were similar. We also show the SBR and SNR comparison of these two probes at different imaging depths. This experiment revealed no substantial differences in SBR or SNR as a function of tissue depth, likely because worms are small and transparent animals.

2. The photostability is a major barrier for red-shifted calcium indicators. What is the highest acquisition rate of recording permitted without significant photobleaching, 2Hz? Can the same neuronal population be imaged repeatedly without significant photobleaching?

Response: We performed additional photobleaching experiments with excitation power intensity that we generally used for imaging at different imaging rates and added the data to Supplementary Fig. 2. Based on these results, 2 Hz seems a reasonably high acquisition rate that is associated with an acceptable rate of photobleaching.

3. In Fig 3, the authors co-expressed HO1 to enhance the BV level in neurons. However, the authors did not mention the fold-increase in terms of brightness without HO1 co-expression.

Response: We co-injected worms with jGCaMP7s and NIR-GECO2 without HO1 and detected no apparent near-infrared fluorescence in neurons of *C.elegans*. We have included the data to the manuscript as Supplementary Fig. 9.

4. It is not clear whether co-expression with jRCaMP or GCaMP6s would influence the expression level of NIR-GECO2/2G. In Fig 3a and 4b, the express patterns of two sensors are not exactly the same. Can authors clarify whether co-expression of another sensor would influence the expression and performance of NIR-GECO?

Response: We performed new experiments and compared the brightness and performance of NIR-GECO2G with and without co-expression of GCaMP6f in neuron cultures. These data suggested that co-expression of GCaMP decreased the brightness of NIR-GECO2G by ~16% but had minimal effect on the dF/F and on/off kinetics of NIR-GECO2G. We have added the results to Fig. 1 and Supplementary Fig. 2.

5. In Fig 3b and Fig 4c, the waveform of jGCaMP7 or GCaMP6s is not exactly the same with that of NIR-GECO2. For example, there is a small transient right before the third puff of NaCl detected by jGCaMP7, but not by NIR-GECO2. Is this due to the sensitivity of the sensor. In Fig 4c, the fluorescence signal of NIR-GECO2G seems to lag behind GCaMP6s. it requires some discussion about the discrepancy of calcium signals detected by different sensors.

Response: This is a valid point. The differences between the waveforms of NIR-GECO2/2G and GCaMP series were probably caused by the slower on-kinetics of

NIR-GECO2/2G and the time-differences when performing two-color imaging (the images were not acquired at exactly the same time due to the switches of two imaging channels back and forth). Nevertheless, we have included the following sentences in the discussion part *"While the off-kinetics of NIR-GECOs are very similar to that of GCaMP6s or jGCaMP7s, the on-kinetics of NIR-GECO series are slower than that of GCaMP series. These slower on-kinetics may mean that closely spaced Ca^{2+} peaks, which could be resolved with a GCaMP variant, will appear to merge into a single peak with NIR-GECO variants. "*

6. As there is no in vivo demonstration in rodent, I assume it remains challenging to image neuronal activity with NIR-GECO2 in rodents. Can the authors discuss the challenges/limitations preventing the use of these variants in rodents?

Response: Indeed, we haven't had much success in obtaining neuronal activity in rodents in vivo at single-cell resolution and we have added the following sentences to the discussion part " *it remains challenging to use NIR-GECO2 and NIR-GECO2G to image Ca^{2+} dynamics with single cell resolution in rodents where neuronal biliverdin concentrations are low and cannot be substantially increased by the strategy of co-expressing HO1 (ref. 8). A promising approach to overcome this challenge was recently described by Kobachi et al., who demonstrated that knocking out the gene for biliverdin reductase increases the biliverdin concentration in mice and improves the brightness of NIR-GECO1 by 4.3-fold*[27]."

7. The authors nicely demonstrated that the sensor can be targeted to nucleus in Fig 3. Though it may not be useful for in vivo imaging, it will be useful to use 2 or 3 colors to detect calcium transients in various subcellular locations. This could be a good discussion point about usefulness of these probes.

Response: Thank you for the suggestion and we have added the following sentence to the discussion part of the manuscript "*With their near-infrared excitation and emission, improved sensitivity, and ability to be subcellularly targeted (e.g., with an NLS), NIR-GECO2 and NIR-GECO2G should prove useful for multi-color and multi-compartment imaging when combined with other fluorescent probes*."
